# Standardized approach to small bowel bleeding in a hierarchical healthcare network with varying levels of complexity

**DOI:** 10.1590/0102-67202025000040e1909

**Published:** 2025-12-08

**Authors:** Rafael Pasqualini de Carvalho, Giovanna Gama-Cunha, Edson Zangiacomi Martinez, José Sebastião dos Santos

**Affiliations:** 1 Universidade de São Paulo, Ribeirão Preto Medical School, Department of Surgery and Anatomy – Ribeirão Preto (SP), Brazil.

**Keywords:** Hemorrhage, Intestine, Small, Capsule Endoscopy, Double-Balloon Enteroscopy, Algorithms, Hemorragia, Intestino Delgado, Endoscopia por Cápsula, Enteroscopia de Duplo Balão, Algoritmos

## Abstract

**Background:**

The systematized approach to patients with small bowel bleeding (SBB) can reduce risks and costs for both patients and the Unified Health System (SUS).

**Aim:**

Evaluate the evolution of the systematized approach to SBB in a regulated, hierarchically organized healthcare network of varying complexity.

**Methods:**

Analysis of the medical records of patients with SBB treated at a tertiary, public, and teaching hospital in two distinct periods: before the implementation of a specialized service and algorithm for SBB (2001–2014, group without algorithm—GSA) and after the establishment of a trained, dedicated team, availability of capsule endoscopy and enteroscopy (2015–2023, group with algorithm—GCA). Demographic, clinical, and care-related data from 184 patient records were collected and entered into the REDCap platform. Additionally, a cost analysis was conducted.

**Results:**

Among the 184 patients, 82 (45%) were in the GSA group and 102 (55%) in the GCA group. The average number of specific exams per patient was 7.19 in GSA and 6.37 in GCA (p=0.02, p<0.05). Blood transfusions were performed in 64 patients (78.05%) in GSA and 68 patients (66.67%) in GCA (p=0.05). The average time to reach diagnosis was 309.9 weeks in GSA and 75.37 weeks in GCA (p<0.01). The average hospital stay was 7.57 weeks in GSA and 2.55 weeks in GCA (p<0.01). In GSA, 19 patients (23.2%) died due to SBB, while in GCA only six did (5.9%) (p=0.001, p<0.05). The average cost was higher compared to GCA (p<0.01).

**Conclusions:**

The results of organizing a reference service for SBB care support are sufficient to subsidize the planning of services and regional healthcare networks.

## ARTICLE HIGHLIGHTS

Obscure gastrointestinal bleeding (OGIB), now referred to as small bowel bleeding (SBB), is characterized by persistent or recurrent blood loss not clarified by conventional upper and lower gastrointestinal endoscopy.

Small bowel bleeding accounts for approximately 5% of gastrointestinal bleeding cases.

High-cost exams, such as capsule endoscopy (CE) and device-assisted enteroscopy, may expedite effective diagnosis, reducing the need for repetitive testing, transfusions, and hospitalizations.

The establishment of a standardized investigation flowchart can improve prognosis and optimize the cost-benefit ratio of diagnostic procedures.

## Summary

**CENTRAL MESSAGE:**

Obscure gastrointestinal bleeding (OGIB), now referred to as small bowel bleeding (SBB), is characterized by persistent or recurrent blood loss not clarified by conventional upper and lower gastrointestinal endoscopy. The difficulty in establishing an etiological diagnosis and providing specific treatment results in frequent repetition of endoscopic and imaging exams, increased need for blood transfusions, prolonged hospital stays, and higher healthcare costs. The incorporation of technological resources and the training of specialists enable the adoption of diagnostic and therapeutic algorithms for patients with SBB.

**PERSPECTIVES:**

The organization of a reference care service for SBB improved the probability of achieving a diagnosis in a shorter timeframe, reduced the need for hospitalization and surgical intervention, and lowered morbidity and mortality rates, thereby offering a better cost-effectiveness ratio. These findings are sufficient to support the planning of services and regional healthcare networks.

## VISUAL ABSTRACT

**Figure:**
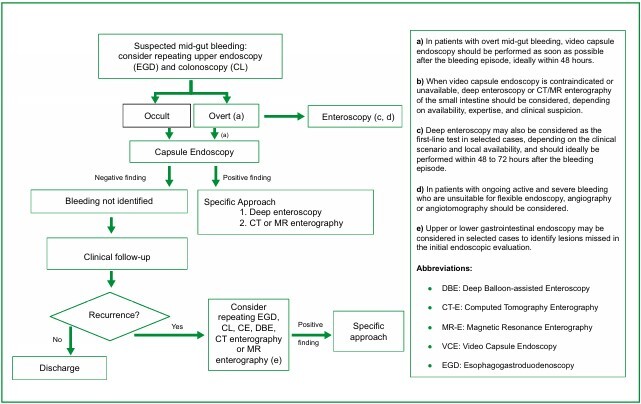

## RESUMO

**Racional:**

A abordagem sistematizada dos pacientes com sangramento digestivo no intestino médio (SIM) pode reduzir riscos e custos para os pacientes e para o Sistema Único de Saúde.

**Objetivo:**

Avaliar a evolução da abordagem sistematizada do SIM em rede assistencial hierarquizada, regulada e de complexidade distinta.

**Métodos:**

Foram analisados os prontuários de pacientes com SIM tratados em hospital terciário, público e de ensino, em dois momentos, antes da implantação de serviço e algoritmo para o SIM, entre os anos de 2001 a 2014 (GSA) e depois mediante definição de equipe capacitada e dedicada, disponibilização da cápsula endoscópica e de enteroscopia e entre os anos de 2015 e 2023 (GCA). Os dados demográficos, clínicos e assistenciais dos prontuários de 184 pacientes foram coletados e inseridos na plataforma redcap. Adicionalmente, fez-se a análise de custos.

**Resultados:**

Dentre os 184 pacientes avaliados, 82 (45%) foram abordados no GSA e 102 (55%), no GCA. O número médio de exames específicos utilizados foi 7,19 por paciente no GSA e 6,37 para o GCA (p=0,02, p<0.05). As transfusões sanguíneas foram realizadas em 64 pacientes (78,05%) no GSA e em 68 pacientes (66,67%) no GCA (p=0,05). A média de tempo para se alcançar o diagnóstico foi de 309,9 semanas no GSA e 75,37 semanas no GCA (p<0,01). A média de tempo de internação foi de 2,55 semanas para o GCA e de 7,57 semanas para o GSA (p<0,01). No GSA, a mortalidade foi de 19 pacientes (23,2%) em decorrência do SIM, enquanto no GCA, apenas 6 (5,9%) (p=0.001, p<0.05). O custo médio foi maior para o GSA, (R$ 44.434,59) em relação ao GCA (R$ 17.818,73) (p<0,01).

**Conclusões:**

Os resultados da organização de referência para atenção ao SIM são suficientes para subsidiar o planejamento dos serviços e das redes regionais de atenção à saúde.

## INTRODUCTION

 Obscure gastrointestinal bleeding (OGIB), now referred to as small bowel bleeding (SBB), is characterized by persistent or recurrent blood loss not clarified by conventional upper and lower gastrointestinal endoscopy. It accounts for approximately 5% of gastrointestinal bleeding cases^6,15^. In the vast majority of cases, the source is located in the small bowel (90%), while in the remaining 10%, the origin is undetected due to technical limitations or invisible lesions during endoscopy or colonoscopy^8^. 

 This scenario presents a significant challenge due to the lack of standardized investigation algorithms, hindering comparison across scientific studies^8,14^. The difficulty in establishing an etiological diagnosis and providing specific treatment results in frequent repetition of endoscopic and imaging exams, increased need for blood transfusions, prolonged hospital stays, and higher healthcare costs^7,14^. Consequently, morbidity increases, with a corresponding rise in mortality rates^2,13^. 

 In clinical practice, numerous care gaps are observed, particularly the absence of clinical and regulatory algorithms for referral, investigation, and treatment of SBB cases. The establishment of an investigation flowchart can improve prognosis and optimize the cost-benefit ratio of diagnostic procedures. High-cost exams, such as capsule endoscopy (CE) and device-assisted enteroscopy, may expedite effective diagnosis, reducing the need for repetitive testing, transfusions, and hospitalizations^8,14^. 

 The incorporation of technological resources and the training of specialists enable the adoption of diagnostic and therapeutic algorithms for patients with SBB. Therefore, it is essential to evaluate the impact of these measures in reducing time to diagnosis and treatment, in healthcare resource utilization, and in costs, as well as in improving patient outcomes. Furthermore, these results can support the development of a clinical and regulatory algorithm to streamline access, diagnosis, and treatment for patients with SBB within a hierarchically organized, regulated, and structurally distinct healthcare network, aiming to achieve better cost-effectiveness in the management of this condition^3^. 

 The initial resistance to prioritize the organization of this reference service at the Clinics Hospital of the Ribeirão Preto Medical School, Universidade de São Paulo, underscores the need for an impact assessment. This can be done through studies analyzing the evolution of clinical and cost indicators, providing evidence to assist decision-makers at public teaching hospitals and health policy managers in the Unified Health System (SUS), in addressing less prevalent health conditions^3^. 

 The objective of this study was to analyze the evolution of care and cost indicators in the systematized management of SBB, within a regulated, hierarchically structured healthcare network of varying complexity. 

 The study was approved by the Ethics Committee of the Institution, Certificates of Presentation for Ethical Appreciation (CAAE) numbers 60218316.8.0000.5440 and 81825824.7.0000.5440. 

## METHODS

 An observational, retrospective and longitudinal study was conducted through the analysis of medical records of patients with gastrointestinal bleeding treated at the Clinics Hospital of the Ribeirão Preto Medical School, Universidade de São Paulo, from 2001 to 2023, comprising a total of 4,857 patients. Only those whose initial presentation included anemia, melena, or hematochezia, and whose endoscopic examinations excluded upper and lower gastrointestinal bleeding, were included. 

 The 184 patients diagnosed with SBB were divided into two groups based on the implementation of a specialized diagnostic and treatment unit at the Endoscopy Center:

- Group without algorithm (GSA): patients treated from 2001 to 2014;
- Group with algorithm (GCA): patients treated from 2015 to 2023.

The collected data included: sex, age, clinical presentation on admission (melena, hematochezia, anemia with occult bleeding), treatment location at symptom onset (emergency room, stabilization unit, inpatient ward, intensive care unit, outpatient clinic), Acute Physiology and Chronic Health Evaluation (APACHE) score, number and types of specific exams performed (computed tomography—CT scan, magnetic resonance imaging (MRI), upper and lower endoscopy, scintigraphy, CE, double-balloon enteroscopy (DBE), interventional therapy, and arteriography), time to diagnosis, number and duration of hospitalizations, complications, transfusions (red blood cells—RBCs and blood products), surgical interventions, final diagnosis, clinical outcome, and cost per patient. 

 For statistical analysis, categorical variables were compared between the GCA and GSA groups using Fisher’s exact test. Quantitative variables were compared using the Wilcoxon nonparametric test. Kaplan-Meier curves were used to describe time to diagnosis, and the Wilcoxon test with correction for ties was applied for group comparisons. Graphs were generated using the "ggsurvfit" package in R. Data organization, statistical analysis, and visualization were performed using R version 4.3.2. A significance level of 0.05 was adopted for all inferential analyses. 

 A micro-costing approach was used to calculate all diagnostic and therapeutic procedures, as well as the costs associated with hospital stays across different care sectors. Each variable was assigned its respective average cost based on 2015 values. The costing method followed the absorption costing model, which includes both direct and indirect costs. For indirect costs, allocation criteria were defined based on the institution’s specific context (technical, human, technological, energy, and material resources)^5^. 

 The incremental cost-effectiveness ratio (ICER) was calculated by dividing the median cost difference of diagnosis and treatment by the difference in effects (i.e., median hospital stay duration and time to diagnosis)^9,17^, between the two groups, defined as Equation 1: 


(1) $$\mathrm{ICER}=\frac{\left[\mathrm{GCA}\text{cost}-\mathrm{GSA}\text{cost}\right]}{\left[\mathrm{GCA}\text{effectiveness}-\mathrm{GSA}\text{effectiveness}\right]}$$

## RESULTS

**Figure 1:**
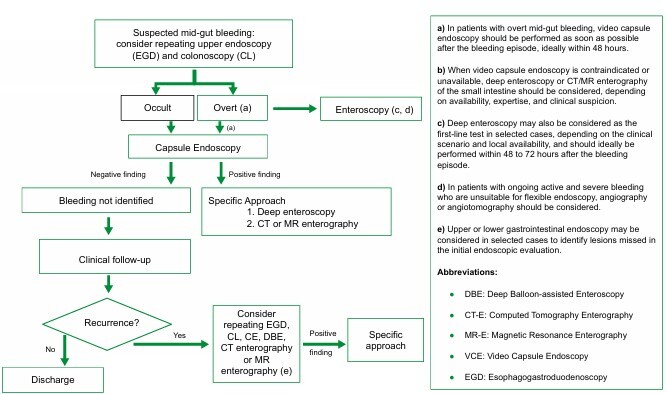
Adapted algorithm for diagnosis and treatment of suspected small bowel bleeding^12^.

 Among the 184 patients diagnosed with SBB, 102 (55.43%) were diagnosed and treated using the defined algorithm (GCA) (Figure 1) and 82 patients (44.57%) were managed without a defined algorithm (GSA). 

**Figure 2:**
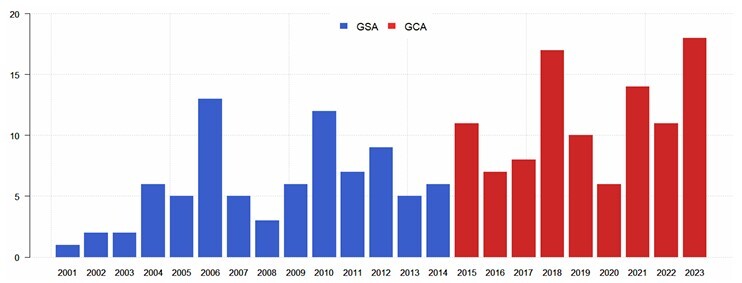
Temporal distribution of cases of small bowel gastrointestinal bleeding according to clinical approach, without algorithm (GSA) and with algorithm (GCA).

 The temporal distribution of patients in each group is shown in Figure 2. 

 The clinical and epidemiological characteristics of patients in the GSA and GCA are presented in Table 1. The clinical interventions and surgical procedures performed are shown in Table 2, while the clinical complications related to SBB are presented in Table 3. The incidences of diagnoses in patients investigated for SBB are shown in Table 4. 

**Table 1:** Epidemiological and clinical characterization of patients with small bowel gastrointestinal bleeding.

		GCA (n %)	GSA (n %)	Total (n)	p-value*
Sex:					
	Female	60 (58.8)	38 (46.3)	98	0.103
	Male	42 (41.2)	44 (53.7)	86	
Age group (Years):					
	Up to 20	14 (13.7)	9 (11.0)	12	0.069
	21 to 40	29 (28.4)	17 (20.7)	31	
	41 to 60	48 (47.1)	24 (29.3)	53	
	61 to 80	3 (2.9)	29 (35.4)	77	
	Over 80	8 (7.8)	3 (3.7)	11	
APACHE Score:					
	≤8	47 (46.1)	23 (28.0)	70	0.015
	>8	55 (53.9)	59 (72.0)	114	
Clinical presentation:					
	Anemia	35 (34.3)	10 (12.2)	45	<0.001
	Hematochezia	26 (25.5)	44 (53.7)	70	
	Melena	41 (40.2)	28 (34.1)	69	
Place of treatment					
	Emergency Room	10 (9.8)	0 (0.0)	10	<0.001
	Clinical Stabilization	1 (1.0)	4 (4.9)	5	
	Inpatient Ward	18 (17.6)	61 (74.4)	79	
	Intensive Care Unit	3 (2.9)	13 (15.9)	16	
	Outpatient	70 (68.6)	4 (4.9)	74	

* Fisher’s exact test was used for all comparisons; GCA: Group with Algorithm; GSA: Group without Algorithm; APACHE: Acute Physiology and Chronic Health Evaluation.

**Table 2:** Interventions performed in patients with small bowel gastrointestinal bleeding.

Type of Intervention:	GCA (n %)	GSA (n %)	Total (n)	p-value*
No intervention	14 (13.7)	0 (0.0)	14	<0.001
Exploratory laparotomy	1 (1.0)	0 (0.0)	1	
Laparotomy + enterectomy	1 (1.0)	0 (0.0)	1	
Laparotomy + enteroscopy	1 (1.0)	0 (0.0)	1	
Thalidomide	1 (1.0)	0 (0.0)	1	
Enteroscopy/argon therapy	8 (7.8)	0 (0.0)	8	
Enteroscopy/clip	1 (1.0)	0 (0.0)	1	
Iron supplementation/supportive therapy	52 (51.0)	57 (69.5)	109	
Combined therapy (≥2 interventions)	23 (22.5)	25 (30.5)	48	

* Fisher’s exact test was used; GCA: Group with Algorithm; GSA: Group without Algorithm.

**Table 3:** Complications related to bleeding in patients with small bowel gastrointestinal bleeding.

Complications:	GCA (n %)	GSA (n %)	Total (n)	p-value*
Bronchopneumonia	5 (4.9)	12 (14.6)	17	0.038
Renal failure	9 (8.8)	9 (11.0)	18	0.628
Anaphylaxis	0 (0.0)	2 (2.4)	2	0.197
Urinary tract infection	9 (8.8)	3 (3.7)	12	0.231
Electrolyte disturbance	0 (0.0)	8 (9.8)	8	0.001
Deep vein thrombosis	2 (2.0)	1 (1.2)	3	1.000
Cardiopathy	5 (4.9)	6 (7.3)	11	0.543
Hepatopathy	0 (0.0)	3 (3.7)	3	0.087
Capsule retention	1 (1.0)	0 (0.0)	1	1.000
Any complication	24 (23.5)	44 (53.7)	68	<0.001

* Fisher’s exact test was used; GCA: Group with Algorithm; GSA: Group without Algorithm.

**Table 4:** Etiological diagnosis of bleeding in patients with small bowel gastrointestinal bleeding.

Diagnosis	GCA (n, %)	GSA (n, %)	Total (n)	p-value*
Undetermined	10 (9.8)	53 (64.6)	63	<0.001
Meckel’s diverticulum	3 (2.9)	1 (1.2)	4	
Inflammatory bowel disease (IBD)	6 (5.9)	3 (3.7)	9	
Angiodysplasia	67 (65.7)	7 (8.5)	74	
Neoplasia	1 (1.0)	5 (6.1)	6	
Other arteriovenous malformations	5 (4.9)	7 (8.5)	12	
Peritonitis	0 (0.0)	1 (1.2)	1	
Non-Meckel diverticula	1 (1.0)	2 (2.4)	3	
Small bowel ulcer	7 (6.9)	3 (3.7)	10	
Small bowel polyps	2 (2.0)	0 (0.0)	2	

* Fisher’s exact test was used; GCA: Group with Algorithm; GSA: Group without Algorithm.

**Figure 3:**
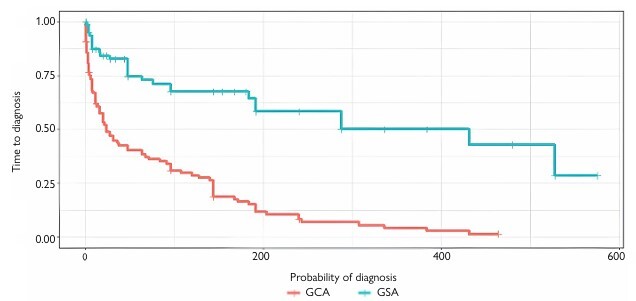
Kaplan-Meier curve correlating probability of diagnosis over time in patients from the groups without algorithm (GSA) and with algorithm (GCA). (Wilcoxon test, p<0.001).

 The main diagnoses found in the GSA group were angioectasias in seven cases (8.54%) and other arteriovenous malformations in another seven (8.54%). In the GCA group, the most frequent diagnosis was angiodysplasia (65.7%) (p<0.001). Diagnosis was not reached in 53 patients (64.6%) from the GSA group and in ten patients (9.8%) from the GCA group. The average time to diagnosis in the GSA group was 309.9 weeks, whereas in the GCA group it was 75.37 weeks (p<0.01) (Figure 3). 

**Figure 4:**
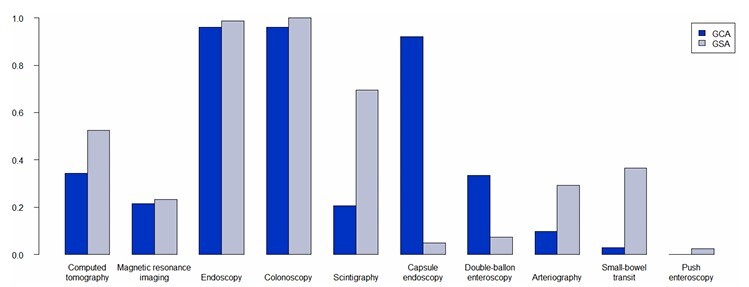
Proportion of diagnostic procedures performed in patients with small bowel gastrointestinal bleeding.

 The proportion of diagnostic procedures performed in patients from the GSA and GCA, such as upper gastrointestinal endoscopy, abdominal CT, magnetic resonance imaging, small bowel transit, labeled RBC scintigraphy, arteriography, push enteroscopy, CE, and DBE, is shown in Figure 4. There was a reduction in the indication of scintigraphy, arteriography, and small bowel transit in the GCA, and an increase in the use of CE and DBE in the same group. 

**Figure 5:**
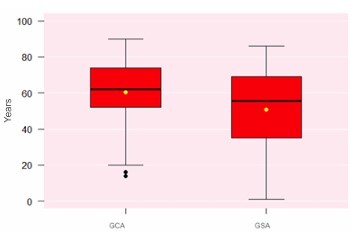
Age distribution of patients with small bowel gastrointestinal bleeding (Wilcoxon test, p<0.01). GCA: Group with Algorithm; GSA: Group without Algorithm.

 Regarding the epidemiological profile of patients affected by small bowel gastrointestinal bleeding (MGIB), the age distributions by group are presented in Figure 5. 

 For patients in the GSA group, the mean age was 50.7 years, while in the GCA it was 60.3 years (p<0.01). 

**Figure 6:**
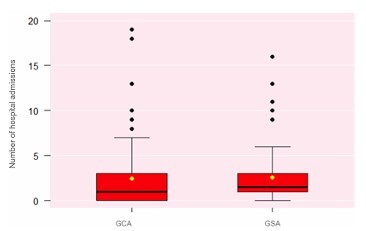
Number of hospital admissions among patients with small bowel gastrointestinal bleeding (Wilcoxon test: p<0.01, p<0.02). GCA: Group with Algorithm; GSA: Group without Algorithm.

**Figure 7:**
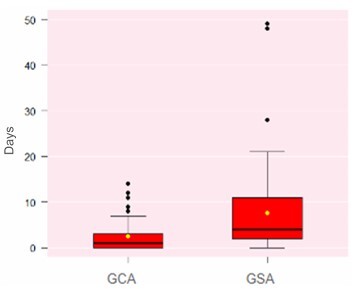
Length of hospital stay among patients with small bowel gastrointestinal bleeding (Wilcoxon test, p<0.01). GCA: Group with Algorithm; GSA: Group without Algorithm.

 A significant reduction was observed in the number of hospital admissions (Figure 6) and in the length of hospital stay (Figure 7) among patients in the GCA. 

**Figure 8:**
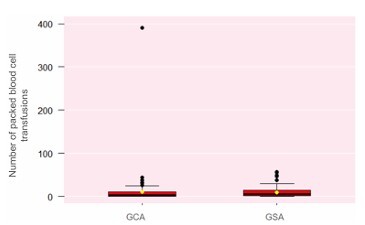
Number of packed red blood cell transfusions per patient with small bowel gastrointestinal bleeding (Wilcoxon test, p=0.05). GCA: Group with Algorithm; GSA: Group without Algorithm.

 The number of packed red RBC transfusions prescribed per patient in the GCA was lower compared to the GSA group, although without statistical significance (Figure 8). 

**Figure 9:**
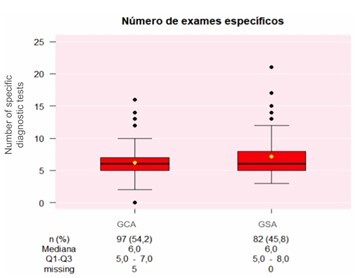
Distribution of the groups according to the number of specific diagnostic tests (Wilcoxon test, p<0.02, p<0.05). GCA: Group with Algorithm; GSA: Group without Algorithm.

 There was a statistically significant reduction in the performance of specific diagnostic tests for investigation of SBB at the GCA (p=0.02, p<0.05) (Figure 9). 

 There was a statistically significant reduction of lethality in the group with the algorithm (GCA) compared to the group without the algorithm (GSA), as shown in Table 5 (p=0.001, p<0.05). 

**Table 5:** Lethality rate during the investigation and treatment of small bowel gastrointestinal bleeding.

Outcome	GCA n (%)	GSA n (%)	Total	p-value*
Survived	96 (94.1%)	63 (76.8%)	159	0.001
Death	6 (5.9%)	19 (23.2%)	25	

* Fisher’s exact test was used; GCA: Group with Algorithm; GSA: Group without Algorithm.

**Figure 10:**
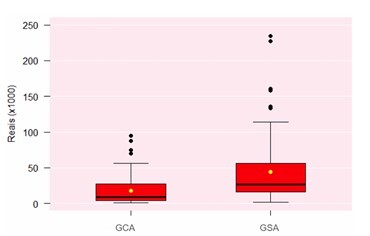
Distribution of the groups according to total cost per patient with small bowel bleeding (Wilcoxon test, p<0.02). GCA: Group with Algorithm; GSA: Group without Algorithm.

 The average cost per patient for the diagnosis and treatment of SBB was R$ 44,434.59 in the GSA, significantly higher than in the GCA, which was R$ 17,818.73 (p<0.02), as shown in Figure 10. 

 Cost-effectiveness calculations demonstrated savings of R$ 5,966.66 for each week of hospital stay avoided, and R$ 380.85 for each week reduced to reach a diagnosis, as shown in the following analysis (Table 6): 

**Table 6:** Incremental cost-effectiveness ratio (ICER) calculation for patients with small bowel bleeding.

Indicator:	GCA	GSA	Difference	ICER (R$)
Median total cost per patient (R$)	8,600.00	26,500.00	-17,900.00	-
Median hospital stay duration (weeks)	1	4	-3	5,966.66
Median time to diagnosis (weeks)	23	70	-47	380.85

ICER: incremental cost-effectiveness ratio; GCA: Group with Algorithm; GSA: Group without Algorithm.

## DISCUSSION

 Although SBB accounts for only 5% of gastrointestinal hemorrhage cases^6,15^, the significant difficulty in the diagnosis and treatment, associated with higher morbidity and mortality rates as well as increased healthcare costs, has encouraged the development of clinical algorithms and reference care teams for investigating this condition. Within the context of Brazil’s Unified Health System (SUS), the Clinics Hospital of the Universidade de São Paulo, as a tertiary and quaternary public referral center, organized a specialized service for the investigation and treatment of SBB^11^. 

 Potential resistance from health administrators to invest in organizing referral services can be mitigated by the clinical and cost-effectiveness outcomes demonstrated in this evaluation. 

 The SBB investigation and treatment service was structured in 2014 by training a specialized team of professionals and by rationally incorporating and applying specific technologies (CE and DBE). The evolution of the approach to SBB across different periods may reflect changes in patient demographic and clinical profiles, potentially introducing assessment bias. However, no significant differences were observed in terms of sex (p=0.103, p>0.05) or age range (p=0.069, p>0.05) among the patients. 

 Prior to the implementation of a standardized approach for SBB, various teams conducted the investigations, often relying on unnecessary and sometimes invasive procedures. This resulted in a lower likelihood of etiological diagnosis and prolonged diagnostic timelines. In this study, the non-algorithm group (GSA) showed an average of 309.9 weeks to reach a diagnosis, with 64.63% of patients remaining without a defined bleeding cause. 

 Additionally, the higher incidence of enterorrhagia (53.7%) observed in the GSA compared to the algorithm group (GCA), where melena and anemia were more frequent presentations (74.5%), may indicate a prolonged bleeding period and increased hematological abnormalities due to delayed diagnosis. 

 In the GSA, a higher incidence (72%) of critically ill patients (APACHE score>8) and older patients (p<0.01, p<0.05) was noted, along with a greater tendency for initial inpatient care in medical wards or intensive care units (90.3%). This contrasted with the GCA, where only 53.9% were critically ill (p<0.015, p<0.05), and hospitalization was required in just 20.5% of cases (p<0.001, p<0.05). 

 International literature recommends a thorough evaluation of the upper and lower gastrointestinal tracts and a complete proctological examination for a comprehensive and systematic assessment of the small intestine. Thus, upon initial referral, the preferred strategy is to repeat upper GI endoscopy and colonoscopy before beginning small bowel diagnostics. It is worth noting that 96% of GCA patients underwent at least one of each of these procedures^4^. 

 The introduction of CE transformed small bowel investigation, allowing a detailed visualization of the distal duodenum, jejunum, and ileum, marking a new diagnostic trend at the hospital^16^. Low-yield procedures such as small bowel follow-through and push enteroscopy were excluded from the diagnostic pathway, while more invasive techniques like arteriography and scintigraphy were employed more selectively and rationally^10,18^. 

 The CE enables ongoing monitoring of gastrointestinal lesions and can evaluate the direct impact of therapeutic interventions^1^. However, limitations exist, especially in detecting lesions in the proximal jejunum or duodenum due to rapid transit. Vascular lesions with predominant extraluminal involvement, subepithelial lesions, small bowel diverticula, and abnormalities in surgically reconstructed intestinal loops may go undetected^11^. Additionally, CE retention may occur in cases of intestinal stenosis, sometimes necessitating surgical removal. 

 DBE proved to be a valuable diagnostic and therapeutic tool, offering sensitivity comparable to CE and serving as a complementary method. In the present study, DBE allowed a more precise identification of bleeding sources and lesions and provided therapeutic options such as clip placement, cyanoacrylate injection, and argon plasma coagulation^11,17,18^. 

 Before algorithm implementation, an average of 7.2 tests per patient was performed, with 64.6% of cases remaining without an etiological diagnosis. Following algorithm adoption, this dropped to 6.5 exams per patient, and undiagnosed cases were reduced to 9.8% (p<0.001, p<0.05). 

 Among patients with a defined diagnosis, the most common lesions were angioectasias in the GCA group (65.7%) and angiectasis and other arteriovenous malformations (8.54%) in the GSA. These findings are consistent with the literature and reinforce that SBB typically presents as anemia and melena, managed on an outpatient basis or in hospital wards when necessary^14^. 

 Regarding the complication rate associated with procedures and hospitalizations, a decrease from 53.7% in the GSA to 23.5% in the GCA was noted (p<0.001, p<0.05). Mortality rates also dropped from 23.2 to 5.9% (p=0.001, p<0.05), and the need for surgical intervention decreased from 30.5 to 5.9% (p<0.001, p<0.05). 

 The mean time to etiological diagnosis was 309.9 weeks in the non-algorithm group and 75.37 weeks in the algorithm group (p<0.01, p<0.05). Nevertheless, these values remain elevated, as newer guidelines recommend CE within two weeks of bleeding^11^. 

 The systematic approach reduced per-patient costs and improved cost-effectiveness by shortening hospital stays and diagnostic times. Both the average cost and its variability were reduced, indicating that monitoring process variability can help evaluate organizational performance and support rational investment in healthcare. 

 The average cost per patient for SBB diagnosis and treatment was R$ 44,434.59 in the GSA and R$ 17,818.73 in the GCA. Median values were R$ 26,500 and R$ 8,600.00, respectively. 

 Given the importance of auditing institutional processes and performance to guide and legitimize the rational use of public healthcare resources, further research is necessary to assess the development of the small intestine reference unit at tertiary reference hospital. 

## CONCLUSIONS

 The organization of a reference care service for SBB improved the probability of achieving a diagnosis in a shorter timeframe, reduced the need for hospitalization and surgical intervention, and lowered morbidity and mortality rates, thereby offering a better cost-effectiveness ratio. These findings are sufficient to support the planning of services and regional healthcare networks. 

## Data Availability

The Informations regarding the investigation, methodology and data analysis of the article are archived under the responsibility of the authors.
